# A role for plasmacytoid dendritic cells in the rapid IL-18-dependent activation of NK cells following HSV-1 infection

**DOI:** 10.1002/eji.200636362

**Published:** 2007-05

**Authors:** Daniel P Barr, Gabrielle T Belz, Patrick C Reading, Magdalena Wojtasiak, Paul G Whitney, William R Heath, Francis R Carbone, Andrew G Brooks

**Affiliations:** 1 Department of Microbiology and Immunology, The University of MelbourneMelbourne, Australia; 1 Division of Immunology, The Walter and Eliza Hall Institute of Medical ResearchMelbourne, Australia

**Keywords:** Cytokines, Dendritic cells, HSV-1, NK cells

## Abstract

Natural killer (NK) cells play a crucial role in the initial response to viral infections but the mechanisms controlling their activation are unclear. We show a rapid and transient activation of NK cells that results in the production of IFN-γ immediately following infection with herpes simplex virus type 1 (HSV-1). Activation of NK cells leading to synthesis of IFN-γ was not mediated by a direct interaction with virus but required the presence of additional cell types and was largely dependent on the cytokine IL-18, but not IL-12. HSV-1-induced IFN-γ expression by NK cells *in vitro* was impaired in spleen cultures depleted of CD11c^+^ cells. Conversely, coculture of NK cells with virus-exposed conventional DC or plasmacytoid (p)DC restored the production of IFN-γ, indicating that multiple DC subsets could mediate NK cell activation. While conventional DC populations stimulated NK cells independently of IL-18, they were less effective than pDC in promoting NK cell IFN-γ expression. In contrast, the potent stimulation of NK cells by pDC was dependent on IL-18 as pDC from IL-18-deficient mice only activated a similar proportion of NK cells as conventional DC. These data identify IL-18 as a crucial factor for pDC-mediated NK cell regulation.

## Introduction

Natural killer cells are critical components of the innate immune system and have key roles in early immune responses to various tumours and viruses 1. While the interaction between NK receptors and ligands expressed on the surface of target cells has been shown to modulate the effector function of NK cells 2, 3 following infection, pro-inflammatory cytokines such as IL-12 and IL-18 are thought to regulate NK cell activation 4–6.

Microbial products such as *Escherichia coli* lipopolysaccharide and unmethylated CpG dinucleotides can induce IL-12 secretion by macrophages and bone marrow-derived DC 7–10. Similarly, distinct lineages of DC isolated from mice can produce IL-12 11. In particular, conventional CD8α^+^ DC and plasmacytoid DC (pDC) populations have been shown to elaborate IL-12 following viral infection or culture with a variety of microbiological stimuli 12–16.

Biologically active IL-18 can be rapidly produced by cells following the proteolytic cleavage of an inactive pro-IL-18 precursor 17, 18, which appears to accumulate within cells such as DC 19. While DC are an important source of IL-18 20, 21, it can also be expressed by a variety of other cells including astrocytes and microglia 22, keratinocytes 23, epithelial cells 24, adipocytes 25 and cells of the adrenal gland and kidneys 26, 27. IL-18 has pleiotropic effects that depend to some extent on the cytokine milieu and acts in concert with IL-12 to induce IFN-γ production by NK cells 28–30.

Numerous studies using neutralising antibodies or animals with targeted gene disruptions suggest that production of both IL-12 and IL-18 is required for control of certain bacterial, fungal, protozoan and viral pathogens in mouse models of infection 31, 32. However, production of both IL-12 and IL-18 during immune responses is not always required. IL-18-deficient mice but not IL-12-deficient mice are susceptible to pneumococcal infection 33. Conversely, while both cytokines collaborate for the induction of splenic NK cell IFN-γ responses following murine cytomegalovirus (MCMV) infection, only IL-12 is required for hepatic NK cell activity 34. Here, we show that splenic NK cells rapidly produce IFN-γ following HSV-1 infection *via* a mechanism largely dependent on the presence of IL-18, but not IL-12. Moreover, while both conventional DC and pDC have the capacity to activate NK cells in response to HSV-1, we identify a role for IL-18 in the potent pDC-dependent stimulation of IFN-γ production by NK cells.

## Results

### The *in vivo* NK cell response following HSV-1 infection is rapid and transient

**Figure 1:**
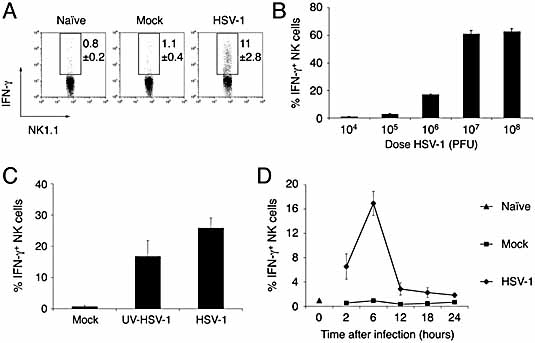
Immediate activation of NK cells following intravenous infection with HSV-1. Spleens were obtained from naïe B6 mice or 6 h after B6 mice were injected intravenously with (A) Vero cell lysate (Mock) or 10^6^ PFU of HSV-1, (B) increasing doses of HSV-1, (C) Vero cell lysate (Mock), 10^6^ PFU of HSV-1 or an equivalent amount of UV-inactivated HSV-1, and the proportion of NK cells producing IFN-γ determined by flow cytometry. (D) Spleens were obtained from naïe, mock-infected (Vero cell lysate) or HSV-1-infected (10^6^ PFU) mice at the indicated times post-infection and the proportion of NK cells producing IFN-γ determined by flow cytometry. Data in panel (A) show representative dot plots of IFN-γ production by NK cells, and have been gated on CD3ε^–^ NK1.1^+^ lymphocytes. Data in panels (B) and (C) show the mean proportion of NK cells producing IFN-γ (± SD) and are representative of three independent experiments. Data in panel (D) show the mean proportion of NK cells producing IFN-γ (± SE) from three independent experiments

Depletion of NK cells prior to infection with HSV-1 can result in elevated viral replication and increased morbidity and mortality 35–37. However, the mechanisms by which HSV-1 infection induces NK cell activation *in vivo* are unclear. We made use of an intravenous model of HSV-1 infection in C57BL/6 (B6) mice to better define the events that result in NK cell activation. Splenocytes isolated from either naïve mice, mock-infected mice or mice infected 6 h earlier with 10^6^ PFU of HSV-1 were cultured for 4 h in the presence of Brefeldin A and the production of IFN-γ by NK cells was assessed by flow cytometry. Few CD3^–^ NK1.1^+^ NK cells from naïe or mock-infected mice expressed IFN-γ (Fig. 1A). In contrast, there was a marked increase in the proportion of NK cells that produced IFN-γ 6 h post-infection with HSV-1. This response was dose dependent, with greater than 60% of splenic NK cells staining positively for IFN-γ following inoculation with 10^7^ PFU (Fig. 1B) but not dependent on viral replication since a similar proportion of activated splenic NK cells was observed when mice were inoculated with UV-inactivated HSV-1 (Fig. 1C).

**Figure 2:**
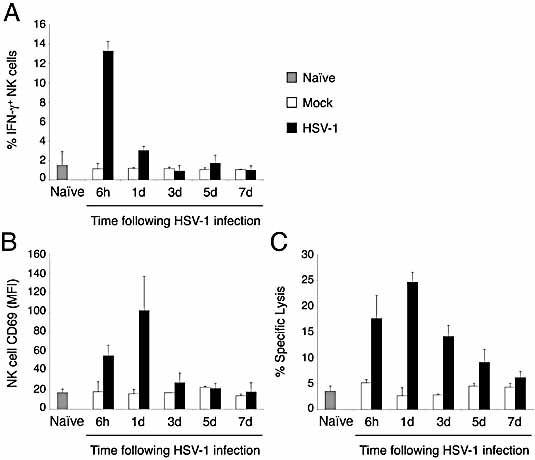
The NK cell response to HSV-1 is characterized by IFN-γ production, CD69 expression and increased YAC-1 cytolysis. Splenocytes were prepared from naïe (shaded column), mock-infected (unfilled columns) and HSV-1-infected (10^6^ PFU, filled columns) mice at 6 h and 1, 3, 5 and 7 days post-infection. Cells were stained with anti-NK1.1-FITC and anti-CD3ε-APC and the expression of (A) intracellular IFN-γ and (B) cell surface CD69 by NK cells was assessed by flow cytometry. (C) Spleen cells were incubated with ^51^Cr-labeled targets at an effector to target ratio of 100:1 and specific lysis was determined. Columns represent mean ± SD of three mice at each time point. Data are representative of two independent experiments.

To better understand the kinetics of NK cell activation following HSV-1 infection, the production of IFN-γ by splenic NK cells was assessed at various times up to 24 h post-infection (Fig. 1D). IFN-γ production was observed as early as 2 h post-infection, peaking at 6 h post-infection before declining to near background levels by 24 h. The kinetics of other parameters that are characteristic of activated NK cells was also assessed. The expression of CD69 and the capacity to lyse the NK cell-sensitive target cell YAC-1 were both increased over naïe and mock-infected controls as early as 6 h post-infection with HSV-1 (Fig. 2). In contrast to the IFN-γ response, maximal cytotoxicity and CD69 expression on NK cells was observed 1 day post-infection. Moreover, while CD69 expression returned to levels observed in naïe mice by day 3, the ability of splenocytes to lyse YAC-1 targets remained elevated until 5 days post-infection (Fig. 2B and C).

### Crucial role of IL-18 for NK cell IFN-γ expression following HSV-1 infection

**Figure 3:**
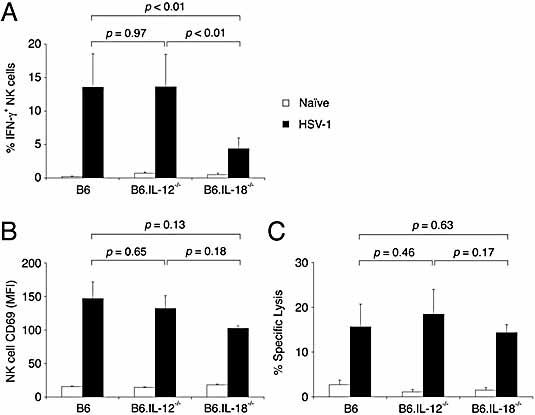
Rapid synthesis of IFN-γ by NK cells in response to HSV-1 infection is dependent on IL-18 *in vivo*. Splenocytes were prepared from B6, B6.IL-12^–/–^ and B6.IL-18^–/–^ mice that were naïe (unfilled columns) or infected (A) 6 h, or (B, C) 24 h earlier with 10^6^ PFU of HSV-1 (filled columns). (A) Cells were incubated for 4 h in Brefeldin A and then stained with mAb specific for NK1.1, CD3ε and IFN-γ. The proportion of IFN-γ^+^ NK cells was subsequently determined by flow cytometry. Columns represent mean ± SD of five mice. Data are representative of at least three independent experiments. (B) Cells were stained with mAb specific for NK1.1, CD3ε and CD69. The expression of CD69 by NK cells was subsequently determined by flow cytometry. Columns represent mean ± SD of four mice. Data are representative of three independent experiments. (C) Spleen cells were incubated with ^51^Cr-labeled targets at an effector to target ratio of 100:1 and specific lysis determined. Columns represent mean ± SE of eight mice from two independent experiments. Statistical significance comparing results from infected animals is indicated.

IL-12 and IL-18 have been shown to activate the synthesis of IFN-γ by NK cells 28–30, 38, 39. Therefore we assessed the contributions of IL-12 and IL-18 to the rapid activation of NK cells following infection with HSV-1 using mice lacking either IL-12p40 (B6.IL-12^–/–^) or IL-18 (B6.IL-18^–/–^). Mice were infected with HSV-1 and 6 h later the production of IFN-γ by NK cells was determined by flow cytometry. NK cells from B6 and B6.IL-12^–/–^ mice responded to HSV-1 infection, with ∼14% of splenic NK cells expressing IFN-γ in both strains (Fig. 3A). This response was significantly reduced in IL-18-deficient mice with ∼4% of NK cells staining positive for IFN-γ protein (*p* <0.001 compared to B6 or B6.IL-12^–/–^ mice).

To further examine the potential roles of IL-12 or IL-18 in the induction of NK cell activation after infection, we also assessed the expression of CD69 by NK cells and lysis of the NK cell-sensitive YAC-1 target cells by splenocytes from cytokine-deficient mice 1 day post-infection with 10^6^ PFU of HSV-1. In contrast to the defect in the production of IFN-γ observed in B6.IL-18^–/–^ mice, the induction of both CD69 and cytotoxicity by NK cells were similar to that observed in both B6 and B6.IL-12^–/–^ mice (Fig. 3B and C). Thus, while IL-12 appeared dispensable for NK cell activation in response to infection with HSV-1, these data demonstrated a role for IL-18 in the immediate and transient production of IFN-γ by NK cells following HSV-1 infection *in vivo*.

**Figure 4:**
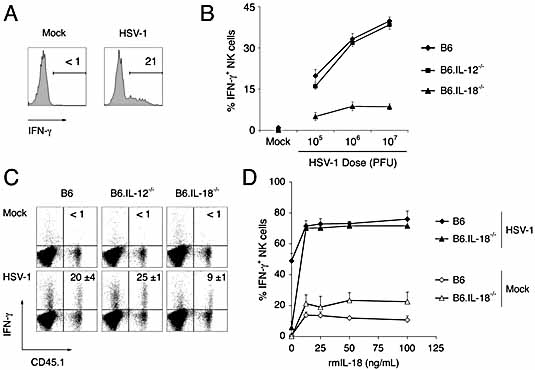
Crucial role of IL-18 for HSV-1-induced IFN-γ production by NK cells. (A) Splenocytes from naïe mice were cultured for 8 h with Vero cell lysate (Mock) or 10^6^ PFU of HSV-1. Cells were then cultured in Brefeldin A for 4 h, stained and the proportion of IFN-γ^+^ NK cells determined by flow cytometry. Representative histograms showing IFN-γ production after gating on CD3ε^–^ NK1.1^+^ cells. (B) The proportion of IFN-γ^+^ NK cells in cultures of B6 (diamonds), B6.IL-12^–/–^ (squares) and B6.IL-18^–/–^ (triangles) splenocytes after incubation with increasing doses of HSV-1. (C) Purified B6.CD45.1 NK cells (5 × 10^4^) were cocultured with 10^6^ CD45.1^–^ B6, B6.IL-12^–/–^ or B6.IL-18^–/–^ splenocytes with Mock inoculum or HSV-1. Values represent the proportion of CD45.1^+^ IFN-γ^+^ events. (D) IFN-γ production by NK cells in B6 (diamonds) and B6.IL-18^–/–^ (triangles) splenocytes induced by Mock inoculum (unfilled) or HSV-1 (filled) in the presence of the indicated concentrations of recombinant mouse (rm)IL-18. (B–D) Data show mean ± SD from triplicate cultures. All data are representative of at least three independent experiments.

To further dissect the mechanisms regulating the rapid and transient production of IFN-γ by NK cells, we assessed the ability of HSV-1 to stimulate NK cells *in vitro*. As observed following *in vivo* infection, the addition of HSV-1 to B6 splenocytes rapidly activated NK cells with ∼21% of CD3ε^–^ NK1.1^+^ cells producing IFN-γ by 8 h (Fig. 4A). The production of IFN-γ was dependent on the addition of virus as few NK cells produced IFN-γ when treated with a mock inoculum that lacked HSV-1. Furthermore, NK cell activation *in vitro* was not dependent on viral replication as the addition of UV-inactivated HSV-1 to splenocyte cultures also stimulated the production of IFN-γ by NK cells (data not shown).

To confirm a role for IL-18 in the rapid induction of IFN-γ production by NK cells, we assessed the ability of splenic NK cells from B6, B6.IL-18^–/–^ and B6.IL-12^–/–^ mice to respond to HSV-1 *in vitro*. Few NK cells produced IFN-γ after culture with a mock inoculum, regardless of genetic background (Fig. 4B). The proportion of NK cells from B6.IL-12^–/–^ splenocytes that produced IFN-γ following the addition of HSV-1 was almost identical to the response observed by NK cells from B6 animals. In contrast, the proportion of NK cells that produced IFN-γ from B6.IL-18^–/–^ splenocytes did not exceed 10% even when 10^7^ PFU of virus was added to cultures.

The poor response of NK cells from IL-18-deficient mice suggested that this cytokine is required for optimal NK cell IFN-γ responses following HSV-1 infection. Alternatively, the impaired response may have resulted from an intrinsic NK cell defect due to their development in the absence of IL-18. To discriminate between these possibilities, congeneically-marked NK cells were purified from B6.CD45.1 mice and mixed with either B6, B6.IL-12^–/–^ or B6.IL-18^–/–^ splenocytes (all CD45.1^–^) and stimulated by the addition of HSV-1 (Fig. 4C). Cells were then stained with anti-CD45.1 mAb and the proportion of CD45.1^+^ cells (*i.e*. wild-type NK cells) expressing intracellular IFN-γ was assessed. In the absence of HSV-1, few CD45.1^+^ NK cells added to CD45.1^–^ splenocyte cultures produced IFN-γ. In contrast, IFN-γ expression was evident in NK cells cocultured with HSV-1 and B6 (∼20%) or B6.IL-12^–/–^ (∼25%) splenocytes. However, there was a marked reduction in the proportion of IFN-γ^+^ CD45.1^+^ NK cells (∼9%) after coculture with IL-18-deficient splenocytes. Thus, the IFN-γ response of B6-derived NK cells to HSV-1 is defective when these cells are cultured with B6.IL-18^–/–^ splenocytes.

We then assessed the ability of exogenous IL-18 to reverse the defective NK cell response observed in IL-18-deficient splenocytes. Again, in the absence of exogenous IL-18, the proportion of IFN-γ-producing NK cells from cultures of IL-18-deficient splenocytes stimulated with HSV-1 was significantly reduced as compared to B6 splenocytes (Fig. 4D). Importantly, the addition of both virus and recombinant IL-18 to splenocytes from B6.IL-18^–/–^ mice restored NK cell activation to similar levels observed for B6 splenocytes. Togethe, the data suggests that the impaired NK cell response in IL-18-deficient animals was not due to NK cell dysfunction but that IL-18 plays a crucial role in the production of IFN-γ by NK cells immediately following HSV-1 infection.

### HSV-1-induced IFN-γ production is not due to direct recognition of virus by NK cells

**Figure 5:**
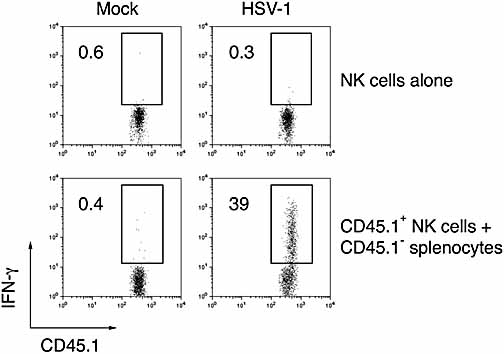
HSV-1-induced activation of NK cells *in vitro* requires other cell types. Purified CD45.1^+^ NK cells (5 × 10^4^) were cultured for 8 h with Vero cell lysate (Mock) or with 10^6^ PFU HSV-1 in the presence or absence of 10^6^ B6 splenocytes (CD45.1^–^). After incubation in Brefeldin A for an additional 4 h, cells were labelled with anti-CD45.1 to identify purified NK cells and then assessed for IFN-γ production. Values represent the proportion of CD45.1^+^ IFN-γ^+^ events and are representative of at least three experiments.

Although the *in vivo* and *in vitro* IFN-γ response of NK cells to HSV-1 occurred with remarkable rapidity, it was markedly dependent on IL-18. This suggested that activation of NK cells was not the result of a direct interaction of NK cells with HSV-1 but rather was dependent on other cell types that could act as a source of IL-18. To confirm that HSV-1-induced IFN-γ production by NK cells required additional cells either as a source of cytokine or to provide crucial cell-to-cell interactions, NK cells were purified from B6.CD45.1 mice (CD45.1^+^) by cell sorting and assessed for their ability to produce IFN-γ following incubation with HSV-1 alone or HSV-1 in the presence of congenically marked splenocytes. While purified NK cells did not respond following the addition of HSV-1, a high proportion of IFN-γ^+^ NK cells were observed following culture with both HSV-1 and congeneic splenocytes (Fig. 5). Therefore, the rapid production of IFN-γ is not induced by a direct interaction between the virus and NK cells but requires the presence of other cell types that may provide crucial cellular interactions or secrete soluble factors including IL-18.

### DC regulate NK cell IFN-γ synthesis in response to HSV-1

**Figure 6:**
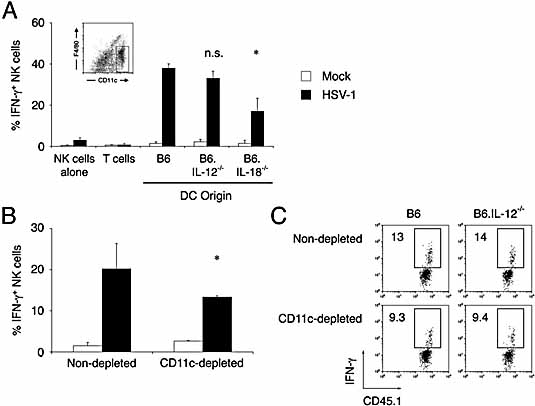
DC-derived IL-18 regulates HSV-1-induced NK cell activation. (A) Splenic DC were purified from B6, B6.IL-12^–/–^ and B6.IL-18^–/–^ mice by cell sorting (insert) and incubated with purified NK cells (1 × 10^4^) with 10^6^ PFU HSV-1 (filled columns) or Vero cell lysate (unfilled columns). Data are shown as mean ± SE. Statistical significance comparing results B6 DC to cytokine-deficient DC is indicated (n.s., not significant; *p 0.05). (B) Splenocytes from B6 mice or splenocytes depleted of CD11c-positive cells were cultured in the presence or absence of HSV-1 and the proportion of NK cells producing IFN-γ determined (mean ± SD) Statistical significance is indicated (*p <0.05). (C) Purified B6.CD45.1 NK cells were cocultured with CD11c-depleted and non-depleted splenocytes from B6 and B6.IL-12^–/–^ mice in the presence of HSV-1 and then stained with anti-CD45.1 and anti-IFN-γ. Values represent the proportion of IFN-γ^+^ CD45.1^+^ NK cells determined by flow cytometry.

Given that HSV-1-dependent stimulation of IFN-γ production required the presence of additional cells found in the spleen, and that a number of DC populations have been implicated in the activation of NK cell responses to viral pathogens 7, 40, we hypothesised that DC could mediate HSV-1-induced NK cell activation. Consequently, we wished to determine if freshly isolated DC could promote NK cell IFN-γ production in response to HSV-1 *via* an IL-18-dependent mechanism. T cells from B6 mice or splenic DC (CD11c^+^ F4/80^–^ low-density cells; Fig. 6A insert) from B6, B6.IL-12^–/–^ and B6.IL-18^–/–^ mice were isolated by cell sorting and assessed for their ability to stimulate IFN-γ production by purified NK cells *in vitro*. Little evidence of NK cell activation was observed following culture of NK cells alone or with purified T cells in the presence of HSV-1 or mock inoculum (Fig. 6A). In contrast, DC from B6 and B6.IL-12^–/–^ mice stimulated ∼38% and ∼33% of NK cells to produce IFN-γ respectively when cultured in the presence of HSV-1. However, there was a significant impairment in the HSV-1-induced activation of purified NK cells following incubation with DC from B6.IL-18^–/–^ mice, with only ∼17% of the cells responding. The data suggest that DC-derived IL-18 is important for the rapid production of IFN-γ by NK cells following HSV-1 infection.

To further confirm a role for DC in mediating the HSV-1-induced production of IFN-γ by NK cells, naïe B6 splenocytes were stained with anti-CD11c mAb and CD11c^+^ cells were depleted from spleen cell preparations using cell sorting. Following 8 h culture in mock inoculum or HSV-1, the proportion of IFN-γ^+^ NK cells in CD11c-depleted cultures was compared to cultures that had been cell sorted without the depletion of CD11c^+^ cells. As shown in Fig. 6B, while few NK cells produced IFN-γ after 8 h culture in mock inoculum, coculture of non-depleted splenocytes with HSV-1 stimulated IFN-γ production by ∼20% of the NK cells. In contrast, the depletion of CD11c^+^ cells from splenocyte cultures significantly reduced the proportion of NK cells that produced IFN-γ in response to HSV-1 to ∼13%.

The CD11c antigen can be expressed on cell types other than DC, including NK cells 41. To ensure that the impaired activation of NK cells in CD11c-depleted spleen cultures was not due to the selective depletion of CD11c^+^ NK cells, purified congeneically marked NK cells from B6.CD45.1 mice were cocultured either with non-depleted or CD11c-depleted splenocytes (both CD45.1^–^) and then stimulated with HSV-1. Cells were then stained with anti-CD45.1 mAb and the proportion of CD45.1^+^ NK cells expressing intracellular IFN-γ was assessed. Again, the percentage of IFN-γ^+^ CD45.1^+^ NK cells after coculture with CD11c-depleted splenocytes was decreased as compared to cultures that contained DC (Fig. 6C).

Whilst these studies confirm a role for DC in the rapid production of IFN-γ by NK cells following exposure to HSV-1, they also highlight the ability of splenocytes of non-DC origin to mediate NK cell activation. No differences were noted in the ability of CD11c-depleted splenocytes from B6 and B6.IL-12^–/–^ mice to induce IFN-γ production by NK cells (Fig. 6C). Together, these data indicate that DC and cells of non-DC origin can activate NK cells after exposure to HSV-1, and that IL-12 is not required for these responses.

### HSV-1-stimulated plasmacytoid DC, but not conventional DC, activate NK cells *via* IL-18

**Figure 7:**
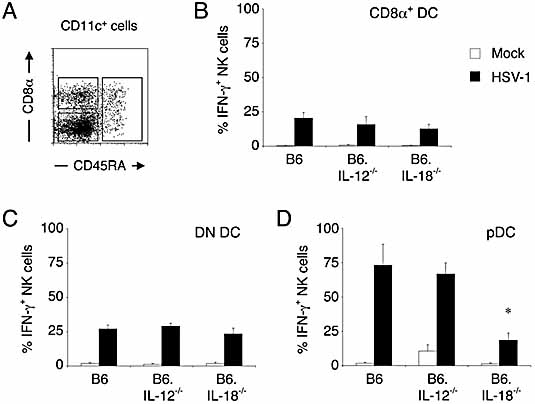
HSV-1-induced NK cell activation is mediated by multiple DC subsets. DC from naïe B6, B6.IL-12^–/–^ and B6.IL-18^–/–^ mice were enriched from splenocytes and stained with mAb specific for CD11c, CD8α and CD45RA. (A) Representative dot plot of CD11c^+^ cells from a B6 animal analysed for expression of CD8α and CD45RA. DC subsets were purified by sorting of CD11c^+^ splenocytes to obtain CD8α^–^ CD45RA^–^ (DN DC), CD8α^+^ CD45RA^–^ (CD8α^+^ DC) or CD45RA^+^ (pDC). Purified NK cells (1 × 10^4^) were incubated with (5 × 10^4^) purified (B) CD8α^+^ DC, (C) DN DC, or (D) pDC subsets in the presence of 10^6^ PFU HSV-1 (filled columns) or Vero cell lysate (unfilled columns) for 6 to 8 h. Following incubation in Brefeldin A for an additional 4 h, cells were stained with anti-NK1.1 and anti-TCRβ. Cells were then fixed, permeabilised and stained for intracellular IFN-γ, and the proportion of NK cells that expressed IFN-γ determined by flow cytometry. The proportion of IFN-γ^+^ NK cells are expressed as mean ± SE from three independent experiments. Statistical significance comparing results from cultures of NK cells mixed with purified cytokine-deficient DC subsets to B6 DC subsets is indicated; *p <0.05.

Numerous studies have demonstrated that there are a number of distinct populations of splenic DC (reviewed in 11). To identify which of these populations played a role in HSV-1-induced activation of NK cells, CD11c^+^ DC were purified from the spleens of B6, B6.IL-12^–/–^ and B6.IL-18^–/–^ animals, separated into subsets based on the expression of CD8α and CD45RA and directly assessed for their capacity to stimulate IFN-γ production by NK cells from B6 mice. DC were sorted into pDC (CD45RA^+^) and conventional DC (CD45RA^–^), the latter of which were further separated into CD8α^–^ (double negative, DN DC) and CD8α^+^ (CD8α^+^ DC) (Fig. 7A). Again, few NK cells produced IFN-γ following culture alone or with purified B6 T cells regardless of whether HSV-1 was present or not (data not shown). Similarly, in the absence of HSV-1, coculture of NK cells with purified DC subsets did not stimulate the production of IFN-γ regardless of genetic background (Fig. 7B–D). In contrast, virus-exposed CD8α^+^ DC obtained from B6 mice stimulated ∼21% of NK cells to synthesize IFN-γ (Fig. 7B). CD8α^+^ DC isolated from either B6.IL-12^–/–^ or B6.IL-18^–/–^ animals stimulated a similar proportion of NK cells to produce IFN-γ in the presence of HSV-1 (Fig. 7B). Purified DN DC from B6, B6.IL-12^–/–^ or B6.IL-18^–/–^ were also equally effective in their ability to stimulate IFN-γ production by NK cells in the presence of HSV-1 (Fig. 7C).

While conventional DC populations from B6 mice stimulated IFN-γ production by ∼25% of NK cells, pDC obtained from B6 mice potently stimulated IFN-γ production by NK cells with ∼73% of NK cells staining positive for IFN-γ (Fig. 7D). The pDC from IL-12-deficient mice stimulated NK cells (∼67%) to levels similar to that observed from B6 animals; however, only ∼19% of NK cells stained positive for IFN-γ after coculture with IL-18-deficient pDC. Thus, in the presence of HSV-1, conventional DC were able to promote IFN-γ production by NK cells *via* an IL-18-independent mechanism. In contrast, the potent ability of pDC to induce IFN-γ production required the presence of IL-18.

## Discussion

The secretion of IFN-γ by NK cells following viral infection is thought to be an important mechanism for limiting the spread of virus prior to the development of adaptive immunity 4. Moreover, recent evidence suggests that IFN-γ expression by NK cells may also be important in the maturation of DC and the induction of a Th1 type response by CD4^+^ T lymphocytes 42, 43. However, the mechanisms by which viral infections induce early IFN-γ production from NK cells are incompletely understood. We have shown that a direct interaction between HSV-1 and NK cells was insufficient to stimulate the rapid production of IFN-γ. Instead, IFN-γ synthesis by NK cells following HSV-1 infection involved regulation by other cell types such as DC or macrophages that could serve as a source of IL-18.

As depletion of DC from *in vitro* splenocyte cultures only partially inhibited NK cell activation, other cells also appear to mediate this function. Consistent with this, removal of F4/80^+^ cells together with CD11c^+^ cells almost totally abrogated the ability of HSV-1 to induce IFN-γ production by NK cells (data not shown), suggesting that DC together with macrophages play a key role in the HSV-1-induced stimulation of NK cells. Critically both DC and macrophages have been shown to express IL-18 6, 20, 21, which has a major role in augmenting the IFN-γ response to HSV-1 by NK cells. Pro-IL-18 is stored in preformed granules within the cytoplasm of cells such as DC or macrophages, and thus may be rapidly converted to its bioactive form 17–19, which may account for its dominant role during the initial stages of HSV-1 infection.

IL-18 has been shown to augment IFN-γ responses by NK cells acting in concert with other cytokines such as type I interferons (IFN-α/β) 44, IL-15, IL-21 45 and in particular, IL-12 28–30. The synergistic action of IL-18 in enhancing IL-12-dependent IFN-γ production by NK cells in the spleen has also been described following systemic MCMV infection in mice 34. However, the production of IFN-γ by NK cells following with HSV-1 infection did not require IL-12, but was largely dependent on IL-18.

Early studies examining cross talk between NK cells and DC identified a role for cell-to-cell contact 42, 46–48. However the mechanisms underpinning the recognition of pathogens and the subsequent induction of innate immune responses by the various lineages of DC is increasingly complex 49. There appears a degree of specialisation in the cytokine responses of particular DC subsets following infection. Following stimulation with HSV-1 or MCMV, pDC produce large quantities of IFN-α/β and IL-12 and hence are thought to be important regulators of NK cells 15, 16. Consistent with this, pDC purified from MCMV-infected mice have been shown to induce both IFN-γ production and cytotoxicity by naïe NK cells 40. We demonstrated that pDC exposed to HSV-1 also efficiently stimulated IFN-γ production by NK cells. Moreover, while the ability of pDC to potently stimulate NK cells has been proposed to result from the high levels of IFN-α/β and IL-12 secreted following viral infection, we define a role for IL-18 in this process. Semino *et al.* 20 have demonstrated that IL-18 is largely confined to the synaptic cleft formed between human DC and NK cells, thus creating localised high concentrations of IL-18. Our inability to detect IL-18 in the culture supernatants of HSV-1-stimulated pDC by ELISA (data not shown) may reflect the polarised delivery of IL-18 from DC to NK cells.

Andoniou *et al* 7 have shown that activation of NK cells by MCMV-infected bone marrow-derived conventional DC is dependent on IL-18 and to a lesser extent IL-12. In our studies using freshly isolated DC populations exposed to HSV-1, there was no evidence of a role for either IL-18 or IL-12 in the activation of NK cells by conventional DC subsets. This apparent discrepancy may be due to (i) subtle differences in the phenotype and function between bone marrow-derived conventional DC cultured *in vitro* and conventional DC freshly isolated from the spleen and/or (ii) differences in the impact of HSV-1 and MCMV on the function of DC 50, 51. As pathogens have evolved a diverse array of strategies to inactivate DC, the evolution of multiple mechanisms to activate NK cells may be an important adaptation to ensure robust early innate immune responses and may even be critical for the generation of adaptive immunity *via* NK cell mediated maturation of DC.

Together, the data indicate a role for IL-18 in the activation of NK cells following HSV-1 infection. While it is likely that a number of distinct cell types can mediate this function, the IL-18-dependent activation of NK cells following HSV-1 infection appears to be unique to pDC among splenic DC populations. Moreover, given recent evidence showing that IL-18 induces the expression of CCR7 on human NK cells 52, pDC may induce lymph node homing of NK cells where they can augment the development of subsequent adaptive immune responses *via* the provision of IFN-γ.

## Materials and methods

### Mice

B6 mice, CD45 congeneic B6.SJL-Ptprc^a^Pep3^b^/BoyJ (B6.CD45.1) mice and mice with targeted gene disruptions of *Il-12p40* (B6.IL-12^–/–^) and *Il-18* (B6.IL-18^–/–^) were housed in the animal facility of the Department of Microbiology and Immunology, The University of Melbourne (Melbourne, Australia). Male mice were used for experiments at 6 to 10 weeks of age. All experimentation was conducted according to institutional ethical guidelines.

### Virus and cytokines

HSV-1 (KOS strain) was propagated and titerd on Vero cells. For *in vivo* infections, mice were injected in the tail vein with 10^6^ PFU of HSV-1 in 200 μL saline. For mock infections, mice were injected intravenously with a freeze thaw lysate of Vero cells in 200 μL saline. For *in vitro* incubations, splenocytes or purified populations of cells were cultured in 200 μL of RPMI supplemented with 10% heat-inactivated FCS, 23.83 g/L HEPES, 2 mM L-glutamine, 50 μM 2-mercaptoethanol, 100 U/mL of penicillin and 100 μg/mL of streptomycin in Vero cell lysate, HSV-1 or recombinant mouse IL-18 (MBL) for 6 to 8 h in round-bottom 96-well plates (TPP).

### Monoclonal antibodies and flow cytometry

Single-cell suspensions were incubated in anti-CD16/32 supernatant (2.4G2) and stained with combinations of peridinin chlorophyll protein-Cy5.5, phycoerythrin-, allophycocyanin- (APC) or FITC-conjugated antibodies to CD3ε (145–2C11), CD8α (53–6.7), CD45.1 (A20), CD45RA/B220 (RA3–6B2), CD49b (DX5), CD69 (H1.2F3), IFN-γ (XMG1.2), NK1.1 (PK136), TCR β chain (H57–597, all BD PharMingen) and F4/80 (BM8, Caltag).

Samples were sorted on MoFlo (DakoCytomation) and FACSAria, acquired on FACSCalibur and LSRII flow cytometers (BD Biosciences) and analysed using FlowJo (Tree Star). Intracellular cytokine staining was performed as described previously 37. For depletion of DC, spleen cells were stained with APC-conjugated streptavidin after incubation in media alone or with biotinylated-anti-CD11c (HL3, BD PharMingen). Cell preparations were then gated on APC-negative splenocytes and purified using cell sorting so that <1% APC-positive cells remained.

### *Ex vivo* NK cell cytotoxicity assay

NK cell cytotoxicity was assayed with a standard chromium release assay using YAC-1 target cells. Percent specific lysis was calculated as 100 × (cpm test sample – cpm spontaneous release)/(cpm total release – cpm spontaneous release).

### Isolation of NK cells and DC

NK cells and T cells were isolated to >95% purity by sorting splenocytes labelled with anti-NK1.1-FITC or anti-CD49b-FITC and anti-CD3ε-APC. DC were isolated from spleens as previously described 53. Briefly, spleens were mechanically disrupted, digested with collagenase type II (Worthington Biochemicals) and grade II bovine pancreatic DNase I (Boehringer-Mannheim), incubated in EDTA and low-density splenocytes were isolated by centrifugation over a Nycodenz gradient (Axis-Shield). For whole DC isolation, cells were then stained with anti-CD11c-FITC (N418) and anti-F4/80-APC and cell sorted to a purity of >90%. For purification of DC subsets, non-DC lineages were removed by incubating cells with mAb specific for CD3 (KT3), Thy-1 (T24/31.7), CD19 (ID6), Gr-1 (RB6–8C5) and erythrocytes (Ter-119), together with sheep anti-rat IgG-coupled magnetic beads (Dynal). Enriched DC were then stained with anti-CD11c, anti-CD8α and anti-CD45RA and sorted by flow cytometry. The purity of DC subsets was typically >95%.

### Statistical analysis

Two-tailed Student's *t*-test was performed where indicated.

## Abbreviations:

APC: allophycocyanin
B6: C57BL/6
MCMV: murine cytomegalovirus
pDC: plasmacytoid DC
